# The role of common genetic variation in presumed monogenic epilepsies

**DOI:** 10.1016/j.ebiom.2022.104098

**Published:** 2022-06-06

**Authors:** Ciarán Campbell, Costin Leu, Yen-Chen Anne Feng, Stefan Wolking, Claudia Moreau, Colin Ellis, Shiva Ganesan, Helena Martins, Karen Oliver, Isabelle Boothman, Katherine Benson, Anne Molloy, Lawrence Brody, The Epi4K Collaborative, Genomics England Research Consortium, Jacques L. Michaud, Fadi F. Hamdan, Berge A. Minassian, Holger Lerche, Ingrid E. Scheffer, Sanjay Sisodiya, Simon Girard, Patrick Cosette, Norman Delanty, Dennis Lal, Gianpiero L. Cavalleri, The Epi25 Collaborative

**Affiliations:** a The SFI FutureNeuro Research Centre, RCSI Dublin, Republic of Ireland; b The School of Pharmacy and Biomolecular Sciences, RCSI Dublin, Republic of Ireland; c Genomic Medicine Institute, Lerner Research Institute, Cleveland Clinic, Cleveland, OH, United States of America; d UCL Queen Square Institute of Neurology, London WC1N 3BG and Chalfont Centre for Epilepsy, Bucks, United Kingdom; e Stanley Center for Psychiatric Research, Broad Institute of Harvard and M.I.T, Cambridge, MA, United States of America; f Division of Biostatistics, Institute of Epidemiology and Preventive Medicine, National Taiwan University, Taipei, Taiwan; g Department of Neurology & Epileptology, Hertie Institute for Clinical Brain Research, University of Tübingen, Tübingen, Germany; h Department of Epileptology and Neurology, University of Aachen, Aachen, Germany; i Axe Neurosciences, Centre de recherche de l'Université de Montréal, Université de Montréal, Montréal, Canada; j Centre Intersectoriel en Santé Durable, Université du Québec à Chicoutimi, Saguenay, Canada; k Department of Neurology, University of Pennsylvania, Philadelphia, PA, USA; l Division of Neurology, Children's Hospital of Philadelphia, Philadelphia, PA 19104, USA; m Department of Biomedical and Health Informatics (DBHi), Children's Hospital of Philadelphia, Philadelphia, PA 19146, USA; n The Epilepsy NeuroGenetics Initiative (ENGIN), Children's Hospital of Philadelphia, Philadelphia, PA 19104, USA; o Epilepsy Research Centre, Department of Medicine, The University of Melbourne, Melbourne, Victoria, Australia; p Department of Medical Gerontology, School of Medicine, Trinity College Dublin, Dublin 2, Republic of Ireland; q Division of Intramural Research, National Human Genome Research Institute, National Institutes of Health, Bethesda, MD, USA; r CHU Sainte-Justine Research Center, Montreal, Quebec, Canada; s Department of Pediatrics, Hospital for Sick Children and University of Toronto, Toronto, Canada; t Department of Neurology & Epileptology, Hertie Institute for Clinical Brain Research, University of Tübingen, Tübingen, Germany; u University of Melbourne, Austin and Royal Children's Hospitals, Melbourne, Australia; v Florey Institute and Murdoch Children's Research Institute, Melbourne, Australia; w Department of Medicine, Neurology Division, Centre Hospitalier de l'Université de Montréal, Montreal, Quebec, Canada; x Department of Neurology, Beaumont Hospital, Dublin, Republic of Ireland; y Epilepsy Center, Neurological Institute, Cleveland Clinic, Cleveland, OH, USA; z Cologne Center for Genomics (CCG), University of Cologne, Cologne, Germany

**Keywords:** Epilepsy, DEEs, PRS, Genetic diagnostics

## Abstract

**Background:**

The developmental and epileptic encephalopathies (DEEs) are the most severe group of epilepsies which co-present with developmental delay and intellectual disability (ID). DEEs usually occur in people without a family history of epilepsy and have emerged as primarily monogenic, with damaging rare mutations found in 50% of patients. Little is known about the genetic architecture of patients with DEEs in whom no pathogenic variant is identified. Polygenic risk scoring (PRS) is a method that measures a person's common genetic burden for a trait or condition. Here, we used PRS to test whether genetic burden for epilepsy is relevant in individuals with DEEs, and other forms of epilepsy with ID.

**Methods:**

Genetic data on 2,759 cases with DEEs, or epilepsy with ID presumed to have a monogenic basis, and 447,760 population-matched controls were analysed. We compared PRS for ‘all epilepsy’, ‘focal epilepsy’, and ‘genetic generalised epilepsy’ (GGE) between cases and controls. We performed pairwise comparisons between cases stratified for identifiable rare deleterious genetic variants and controls.

**Findings:**

Cases of presumed monogenic severe epilepsy had an increased PRS for ‘all epilepsy’ (p<0.0001), ‘focal epilepsy’ (p<0.0001), and ‘GGE’ (p=0.0002) relative to controls, which explain between 0.08% and 3.3% of phenotypic variance. PRS was increased in cases both with and without an identified deleterious variant of major effect, and there was no significant difference in PRS between the two groups.

**Interpretation:**

We provide evidence that common genetic variation contributes to the aetiology of DEEs and other forms of epilepsy with ID, even when there is a known pathogenic variant of major effect. These results provide insight into the genetic underpinnings of the severe epilepsies and warrant a shift in our understanding of the aetiology of the DEEs as complex, rather than monogenic, disorders.

**Funding:**

Science foundation Ireland, Human Genome Research Institute; National Heart, Lung, and Blood Institute; German Research Foundation.

**Research in context**

**Evidence before this study**

We searched the pubmed database from inception until August 30^th^ 2021 for studies related to this research. Search strategies combined the terms ‘epileptic encephalopathies’, OR ‘developmental and epileptic encephalopathies’, ‘epilepsy’, ‘monogenic disease’, and ‘polygenic risk scoring’. No language restrictions were applied. We found high quality studies examining the role of polygenic risk in the complex epilepsies, and in broad developmental disorder phenotypes. Research into the genetics of developmental and epileptic encephalopathies exclusively examined rare genetic variation, from either sequence or CNV data. No studies had examined the role of common genetic variation in the developmental and epileptic encephalopathies (DEEs).

**Added value of this study**

To our knowledge this is the first study of polygenic burden in the DEEs. Using data from six cohorts of severe epilepsies we show that DEEs and similar forms of epilepsy which present with intellectual disability have an increased burden of polygenic risk associated with complex forms of epilepsy. We co-analyse polygenic burden with rare variant data and observe no difference in polygenic burden between cases with and without deleterious rare variants.

**Implications of all the available evidence**

We provide evidence of a polygenic contribution to the DEEs. This study motivates future research of the DEEs as more complex, rather than purely monogenic, disorders. To fully understand the genetic aetiology of the DEEs, studies should incorporate data from microarrays in addition to exome sequencing, or move towards whole-genome sequencing.

Alt-text: Unlabelled box

## Introduction

Developmental and epileptic encephalopathies (DEEs) are a devastating group of epilepsies, characterised by severe epilepsy and developmental slowing or regression associated with epileptiform activity on electroencephalography (EEG).^1^ Individuals with DEEs have intellectual disability (ID), and tend not to have a family history of epilepsy.^2^ DEEs without an obvious acquired cause are now known to be often due *de novo* dominant genetic variants of large effect, although autosomal recessive and X-linked forms are also recognised.^3^^,^^4^ With current technology, genomic testing of people with DEEs, or other forms of epilepsy with ID, can provide a genetic diagnosis in up to 50% of cases.^5^, ^6^, ^7^, ^8^, ^9^, ^10^

The additive effects of common and rare genetic variation have been examined in several neurological disorders. The Deciphering Developmental Disorders (DDD) study used a method known as linkage disequilibrium score regression (LDSC^11^) to estimate that 7.7% of the phenotypic variance of a broad, presumed monogenic, developmental disorder phenotype is attributable to common variants (SNP-based heritability *h^2^* = 7.7%). Polygenic risk score (PRS) analysis is a method that quantifies an individual's burden of common genetic risk variants.^12^^,^^13^ A separate DDD study used PRS to show that a common genetic burden for a broad neurodevelopmental phenotype was present in children with and without identifiable deleterious rare genetic variants.^14^ It has been hypothesised that a neurodevelopmental genetic risk burden could modulate the clinical presentation of neurodevelopmental phenotypes, explaining why disease-causing genetic variants have variable penetrance in different patients.^15^ The interplay between common and rare genetic variation is under increasing focus in other complex neurological conditions. For example, polygenic burden for schizophrenia has been shown to be elevated in people with schizophrenia both with and without deleterious copy number variants (CNVs^16^). However, this polygenic burden appears to be higher in schizophrenia patients without identifiable deleterious CNVs.^17^ In autism, PRS have been shown to confer additive risk to individuals with damaging rare variants.^18^

The common epilepsies have a complex aetiology. Genetic variation, both common,^19^^,^^20^ and rare,^21^^,^^22^ as well as environmental impact^23^ are known contributing factors to epilepsy development. PRS have previously been shown to distinguish individuals with complex epilepsies, both focal and generalised, from controls.^20^ Research into the genetic bases of DEEs has focused on rare-variant data, generated from exome sequences.^21^^,^^22^ Currently, the contribution of common genetic variation to the DEEs remains unclear.

Here we aimed to examine the role of polygenic burden in the DEEs and epilepsies with ID. Using PRS derived from the largest GWAS to date of the common epilepsies,^19^ we compared individuals with 1) DEEs, and 2) epilepsies with ID, to population controls. We then combined our results with rare variant data from available exome and whole-genome sequences to determine whether PRS differed among cases with and without identifiable likely deleterious or pathogenic rare genetic variants.

## Methods

All research participants or their legal guardians provided written, informed consent using protocols approved by ethics committees at each study site.

### Cohort and data descriptions

Genetic and phenotype information were obtained from the following six studies on epilepsy and related neurodevelopmental disorders. Three of the resulting cohorts were DEE (Epi25, Epi4K and CENet) and three were epilepsy with ID (DDD, Irish Lighthouse and GEL). We included ‘epilepsy with ID’ in addition to DEE as, from the clinical perspective, they are considered very similar, there is a high degree of overlap in the causative genes^24^ and the diagnostic yield from genomic testing is comparable.^5^^,^^25^

**Epi25:** Singleton-based microarray, exome, and phenotype data on DEE patients were acquired from the Epi25 collaborative (http://epi-25.org/), an international project aiming to generate sequence data on 25,000 people with various forms of epilepsy. Details on phenotype and exome sequence generation are described elsewhere,^21^ as are details of microarray analysis.^20^ These data were used in combination with control data from the Mass General Brigham (MGB) Biobank (previously the Partner's Healthcare Biobank^26^) following previously described methodology.^20^

**Epi4K project:** Focused on the genetics of epilepsy, the Epi4K collaboration has generated exome data on over 4,000 people with various types of epilepsy.^27^ Previously published microarray data on affected probands, and exome data on DEE trios were obtained from the Epi4K collaborative.^3^ These were analysed with control data from the Australian QSkin study.^28^ Samples in Epi4K which were also in the Epi25 dataset were removed prior to analysis.

**Canadian CENet cohort:** The Canadian Epilepsy Network (CENet) is a Canadian, trio-based study into the genetics of severe epilepsies. Whole genome sequence (WGS), microarray, and phenotype data of DEE patients and their parents were obtained from CENet. WGS data was also available on both of each proband's parents and used to identify likely pathogenic variants, and also allowed for polygenic transmission disequilibrium testing (pTDT). Full details on WGS generation and analysis for this cohort have been described previously,^29^ as have details on microarray genotyping and analysis.^30^

**Deciphering Developmental Disorders study (DDD):** The DDD project is an exome and microarray-based study into the genetic bases of presumed monogenic developmental disorders.^14^ We identified and included the subset of samples in the DDD study with seizure disorders, using the HPO anthology term for ‘Seizures’ and all downstream HPO terms (HP:0001250). Data from the UKBiobank were used as controls after screening for European ancestry (UKBiobank data field 22006) and removing any samples with epilepsy, using available ICD coding.^31^

**Irish Epilepsy Lighthouse:** The Irish Epilepsy Lighthouse is an Irish research project investigating aiming to provide genetic diagnostics for children and adults with epilepsy and ID. Trio-based microarray, exome and phenotype data on children and adults with epilepsy and ID were acquired from the Irish Epilepsy Lighthouse study. Details of phenotype and of exome data generation have been published elsewhere.^5^ We supplemented this data with microarray data generated of the probands using the Illumina Global Screening Array chip. The Irish Epilepsy Lighthouse genotype data were analysed alongside genotype data from the Irish DNA Atlas^32^ and the Trinity Student Dataset,^33^ which were used as controls in the PRS case-control analysis.

**Genomics England (GEL):** WGS data were accessed on the Genomics England research environment, containing genetic data from the UK National Genomics Research Library.^34^ The epilepsy with ID cohort comprised cases with an established diagnosis of epilepsy, confirmed by a neurologist and with at least one additional phenotype from intellectual disability, autism spectrum disorder, structural abnormality (e.g. dysmorphism, cerebral or somatic malformation) and/or unexplained cognitive/memory decline. Controls were taken from the Genomics England renal and urinary tract disorders disease group, using the Genomics England research environment graphical user interface (GUI) to exclude any patients with syndromes with prominent renal abnormalities.

### Imputation and quality control

All data generated from microarrays underwent the following imputation and quality control (QC) process. Datasets which were genotyped on different microarrays were processed separately prior to imputation. QC was conducted using PLINK 1.9, unless otherwise specified.^35^ Pre-imputation, SNPs were removed if present in <98% of samples, if minor allele frequencies (MAFs) were <1%, or Hardy-Weinberg equilibrium (HWE) deviation *P*-values were <10^−5^. Samples were removed if SNP coverage was <98%. For each cohort, samples were screened for European ancestry by merging with data from the Human Genetic Diversity Project,^36^ or 1000 Genomes Project V3,^37^ thinning for linkage-disequilibrium (plink –indep-pairwise 1000,100,0.1), and calculating the top two genetic principal components based on a variance-standardised relationship matrix as implemented in PLINK. The PCs were then plotted using the ggplot2 program in R v3.5,^38^ and the resultant PCA plots were visually inspected to ensure all samples were of European ancestry. Per-sample genotype heterozygosity was plotted and any samples with high levels of genomic heterozygosity were removed. Genotypes were pre-phased using EAGLE v2.4.1.^39^ Imputation to the HRC1.1 imputation panel^40^ was performed for data hosted in Europe (Irish Lighthouse, DDD) using PBWT ^41^ as implemented on the Sanger Imputation Server, or Minimac4 as implemented on the University of Michigan Imputation Server,^42^ for data hosted in outside Europe (Epi25, Epi4K, CENet). Post-imputation, SNPs with imputation INFO scores >=0.9 (or rsq scores >0.3 if imputed using the Michigan server) were kept, and case and control cohorts were then merged.

To ensure genetic homogeneity within each analytical grouping, the top two genetic PCs were calculated using PLINK and plotted, and any outlying samples on the PCA graph were removed from further analysis (Supplemental Figure S1). SNPs with >98% coverage, MAFs>1%, and HWE deviations *P*>10^−5^ were kept.

Genotype data from GEL were generated from WGS rather than microarrays, and as such, no imputation was required.

### Qualifying rare variant analysis

Samples from each epilepsy cohort were divided into those that contained an identifiable predicted damaging variant (‘screen-positive’), and those that did not (‘screen-negative’).

The criteria for ‘screen positive’ differed among cohorts, depending on data availability.

**CENet:** Variant pathogenicity was assigned based on analysis of trio WGS data, with input from the patient's clinical team as previously described.^29^

**Irish Epilepsy Lighthouse:** Variant pathogenicity assigned based on trio analysis of exome sequence and array-CGH data, using interpretation guidelines set out by the American College of Medical Genetics and Genomics.^43^

**Epi4K:** The screen-positive group was composed of carriers of a de-novo protein-altering (missense or null) variant in an established or candidate epilepsy-associated gene or a de novo pathogenic copy number variant as previously described.^44^

**GEL**: The Genomics England Rare Disease tiering process was used to annotate variants that are plausibly pathogenic, based on their effect on protein coding, segregation in the family (where possible), frequency in control populations, mode of inheritance, and whether they are in a gene in the virtual gene panels applied to the family.^45^ The phenotypes of ∼320 patients with pre-selected Tier1/2 variants were discussed at multi-disciplinary meetings with epileptologists, clinical geneticists and laboratory scientists, with a consensus agreement on the pathogenicity of the tiered variants using ACMG criteria.^43^

**Epi25 and DDD:** We bioinformatically inferred likely damaging variants in the Epi25 and DDD cohorts. Deleterious CNVs were those which were >2Mb in length, overlapped with known epilepsy genes or hotspots, or overlapped with a gene with a protein loss-of-function intolerance score (pLI) >0.9.^46^^,^^47^ Variant annotation was performed in ANNOVAR. Deleterious variants were restricted to known, dominant acting epilepsy and ID genes.^48^^,^^49^ Adapting variant classification protocol previously used by Epi25,^21^ deleterious variants were required to be either loss-of-function or missense variants with MPC (Missense badness, PolyPhen-2, and Constraint) scores >2.^46^ Variants were excluded if they appeared in a given dataset >3 times. Likely damaging variants were required to be absent from population databases.^50^

### PRS calculation and statistical analysis

PRS were calculated using the summary statistics of the ILAE 2018 genome-wide association study (GWAS) in complex forms of epilepsy.^19^ Three epilepsy subtypes were used for PRS calculation; ‘genetic generalised epilepsy’ (GGE), ‘focal epilepsy’, and ‘all epilepsy’. ‘All epilepsy’ refers to all cases of epilepsy considered in the ILAE 2018 GWAS paper, which included the focal and GGE cohorts, with a small number of unclassified cases.^19^ PRS for an unrelated ‘control’ phenotype were also calculated in each cohort (Supplemental Table S1). SNPs with *P*-values ≤0.5 from these GWASs were included in the PRS calculation. Statistical analyses of the data were carried out in R v3.5.^38^ To avoid sample overlap, PRS for the Irish Epilepsy Lighthouse study were derived from the ILAE epilepsy GWAS,^19^ recalculated after removing the Irish case and control cohorts (ILAE cohort names: ‘Dublin’, ‘TCD controls’). PRS in the GEL dataset were generated from the ILAE epilepsy GWAS^19^ recalculated after removing the cases from University College London (ILAE cohort name: ‘UCL’). In each analysis cohort, PRS were normalised across all samples to mean 0 and standard deviation 1 and regressed onto phenotypes. The glm() function in R was used to generate a binomial linear regression model, and estimate β-coefficents and standard errors of PRS each model. Each samples’ sex and the top four PCs were included as covariates in each analysis. Nagelkerke's pseudo-*R^2^* was calculated in each cohort as a measure of variance explained by PRS. Each of the three epilepsy PRS models were then meta-analysed across cohorts using a fixed-effects weighted estimate model, as implemented in the rma.uni() function from the ‘metafor’ R package,^51^ which also produced heterogeneity measures (*I^2^*) for each model. As a control, PRS for an unrelated trait were calculated and compared between cases and controls in each analysis cohort (Supplemental Table S1). As a comparison, metafor's rma.uni() function was also used to estimate random-effects meta-analysis models for each PRS.

We then split out cases in each cohort into those with and without an identifiable, likely damaging genetic variant (‘screen-positive’ and ‘screen-negative’, respectively, see above) and performed a multinomial pairwise comparison of each epilepsy PRS between controls, ‘screen-positive’ cases, and ‘screen-negative’ cases, using the multcomp R package.^52^ These comparisons were then meta-analysed across cohorts. Data visualisation was done using ggplot2 v 3.3.3^53^ in R.

### pTDT testing

The CENet cohort contained GWAS genotype data from each individual's parents. Polygenic transmission disequilibrium testing (pTDT^18^) is a method that performs PRS analysis in trios to show an eventual over-transmission of risk alleles. pTDT was analysed following the approach by Weiner et al. based on individual PRS of parents and offspring for all three epilepsy subtypes. pTDT was calculated for the entire cohort, and split into ‘screen-positive’ and ‘screen-negative’ cases.

### Role of the funding source

The funders had no role in study design, data collection, data analyses, interpretation, or writing of this report

## Results

### Case and control descriptions

In total, we analysed 2,759 cases and 477,760 controls, split across 11 cohorts. 460 people carried a variant of likely large effect (‘screen-positive’), and 2,109 did not (‘screen-negative’). A small portion of cases (n=190) in the CENet and Epi4K datasets lacked rare variant data, and as such could not be assigned as either screen-positive or screen-negative. A full breakdown of case and control numbers is shown in Table 1. Samples in the Epi25, Epi4K and CENet cohorts had a neurologist-confirmed diagnosis of a DEE. The Irish Lighthouse, DDD, and GEL cohorts had ‘epilepsy with ID’, although many cases in these cohorts would have also had true DEEs, and would have a phenotype which warranted exome sequencing or WGS to investigate a potential monogenic cause.

**Table 1:** Case and controls numbers per cohort of all samples included in analysis. ‘Screen-positive’ and ‘screen-negative’ indicate the subset of cases in each cohort that did or did not contain an identifiable likely damaging genetic variant, respectively (see Methods: qualifying rare variant analysis). Cohorts which were paired for analysis are grouped by colour.

Cohort	Epilepsy	Screen-positive	Screen-negative	Controls	Phenotype	Data types
Epi25	1,094	163	931	210	DEE	Microarray + exome
Partner's Biobank	0	0	0	19,762	Controls only	Microarray
Epi4K	266	44	77	0	DEEs	Microarray + exome
QSkin	0	0	0	15,717	Controls only	Microarray
CENet	171	40	86	0	DEE	WGS + microarray
Canadian Controls	0	0	0	6,901	Controls only	Microarray
DDD	897	152	745	0	Seizures + ID	Microarray + exome
UK Biobank	0	0	0	400,835	Controls only	Microarray
Irish Lighthouse	82	29	53	0	Epilepsy + ID	Exome (trios) + microarray (probands)
Irish Controls	0	0	0	2,404	Controls only	Microarray
Genomics England	249	32	217	1,931	Epilepsy + ID and controls	WGS
Total	2,759	460	2,109	447,760		

### Elevated epilepsy PRS in severe epilepsy cases relative to controls

#### Additional genetic contributions to apparently monogenic epilepsies

**Figure 1:**
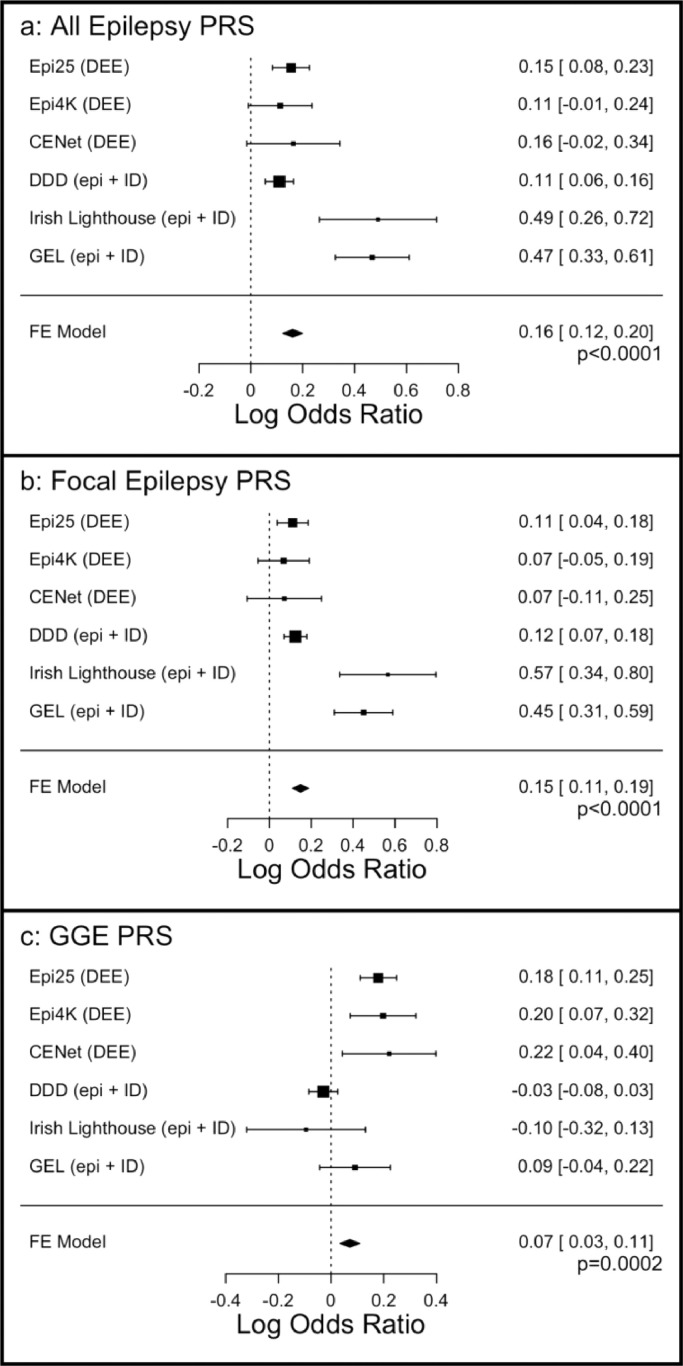
Meta-analysis of PRS of a) ‘all epilepsy’, b) ‘Focal epilepsy’, and c) GGE. ‘FE Model’ = Fixed-effects model. Box plots show log odds ratios and standard errors.

To determine whether epilepsy PRS are increased in severe epilepsies relative to the general population, we calculated PRS in each analytical cohort and incorporated them into a fixed-effects meta-analysis model. Meta-analysis of all analytical groups showed a significant increase in PRS for ‘all epilepsy’ (p<0.0001), ‘focal epilepsy’ (p<0.0001), and ‘GGE’ (p=0.0002) in epilepsy cases relative to controls (Figure 1). Results remained significant when run using a random effects model (see Supplemental Figure S2). Differences in the strength of PRS association were observed between cohorts. PRS for ‘all epilepsy’ and ‘focal epilepsy’ did not significantly distinguish cases from controls in the Epi4k or Canadian CENet cohorts. Contrastingly, PRS analysis for ‘GGE’ did not reach statistical significance in the DDD, Irish Lighthouse, or Genomics England cohorts, all of which had a broader ‘epilepsy with ID’ phenotype, rather than exclusively DEE (Figure 1). The variance explained by each of the PRS can be found in Table 2, and heterogeneity scores in Supplemental Figures S3 and S4.

**Table 2:** Variance explained by each epilepsy PRS in each group of cases. Grey cells indicate analyses that were not statistically significant (see methods: PRS calculation and analysis).

Cohort	All Epi *r*2	Focal Epi *r^2^*	GGE *r^2^*
**Epi25**	1.6%	1.2%	1.5%
**Epi4K**	-	-	0.35%
**CENet**	-	-	1.2%
**DDD**	0.08%	0.1%	-
**Lighthouse**	2.6%	3.3%	-
**Genomics England**	2.8%	2.6%	-

Given the high levels of heterogeneity observed, we re-ran the meta analysis separately for the DEE and the epilepsy plus ID cohorts. Results from DEE-only cohorts (i.e. Epi25, EPI4k and CENet) showed that all PRS remain statistically significant (‘all epilepsy’ PRS p<0.0001, ‘focal epilepsy’ PRS p=0.0016, and ‘GGE’ PRS p<0.0001, with low heterogeneity (I^2^ = 0, see Supplemental Figure S3). Results from Epilepsy Plus ID-only cohorts (i.e. DDD, Irish Lighthouse and GEL) showed significance for ‘all epilepsy’ PRS (p<0.001), and ‘focal epilepsy’ PRS (p<0.001), but not GGE (p=0.51), with considerable heterogeneity (I^2^ ranges: 36-93%) see Supplemental Figures S3 and S4.

#### Elevated PRS in the presence of highly deleterious rare genetic variants

**Figure 2:**
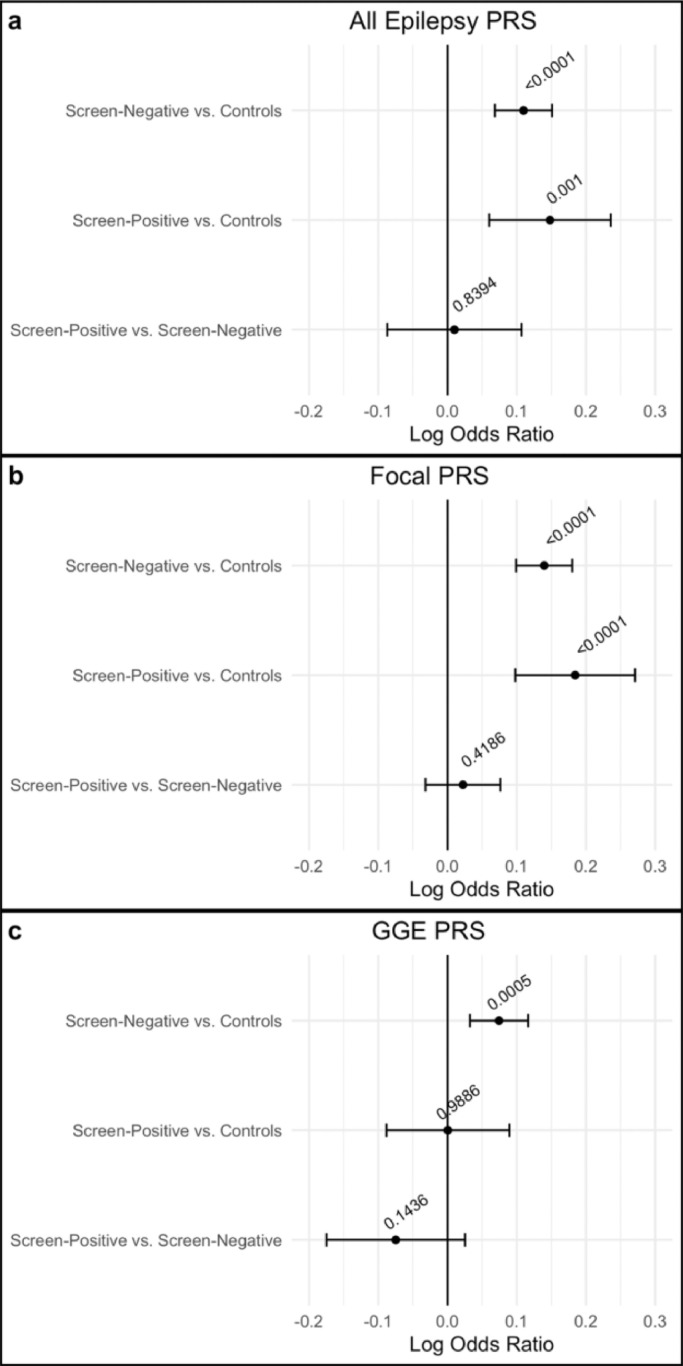
Fixed-effects meta-analyses comparing cases with or without likely deleterious genetic variants to each other and population controls for a) ‘All epilepsy’ PRS, b) Focal PRS, and c) GGE epilepsy PRS. Log odds ratios and 95% confidence intervals are displayed in the error bars. P-values for each model are shown as numbers.

We next aimed to delineate epilepsy PRS between cases based on the presence of a rare damaging variant by splitting our cases in each cohort into screen-positive’ and ‘screen-negative’ and comparing each of the three epilepsy PRS between screen-positive cases, screen-negative cases, and controls. Meta-analysis of these PRS across all study cohorts showed that both screen-positive and screen-negative cases had an elevated PRS for ‘all epilepsy’ and ‘focal epilepsy’ relative to controls (Figure 2). For GGE PRS we observe an increased PRS in screen-negative cases relative to controls, but no significant difference between screen-positive cases and controls. We did not find any significant differences in epilepsy PRS between screen-positive and screen-negative cases (Figure 2, Table S2).

**pTDT:** The presence of parental genotype data in the CENet cohort allowed for pTDT analysis, which has the advantage of removing any genetic population structure as a potential confounder.^18^ Significant enrichment was seen for ‘GGE’ PRS in affected offspring relative to parents (*P*= 4.46×10^−9^). This signal maintained significance when cases were split into ‘screen-positive’ and ‘screen-negative’ (*P*=2.57×10^−2^, *P*=7.10×10^−4^, respectively). PRS analyses for ‘all epilepsy’ and ‘focal epilepsy’ did not meet the threshold for statistical significance. These results support our previous results from the case/control analysis in CENet, which found a significant elevation for GGE PRS in cases relative to a different group of normal controls.

## Discussion

We calculated epilepsy PRS in a cohort of 2,759 patients with DEEs or severe epilepsies and analysed them together with 447,760 population controls. We observed an enrichment of epilepsy PRS in patients with DEEs and ‘epilepsy with ID’. In the CENet dataset we also observe an over-transmission of GGE PRS from parents to DEE affected offspring in pTDT analysis. Thus, we present evidence that common genetic variation plays a role in the aetiology of the DEEs and other forms of ‘epilepsy with ID’.

We found that PRS for ‘all epilepsy’ and ‘focal epilepsy’ were increased in both patients with a known pathogenic variant of major effect (screen-positive) and those without (screen-negative), while GGE PRS was only increased in screen-negative cases. We did not observe a significant difference in any PRS between screen-positive and screen-negative patients. Our results complement those of a recent study of DEE that demonstrated an enrichment of damaging ultra-rare variants in non-EE/DEE genes, even in the subset of cases where a diagnostic variant had previously been identified.^54^ In combination, these results suggest the DEEs as a group of disorders with key diagnostic mutations acting on a background of complex genetic architecture. Larger samples sizes and improved diagnostic yield are needed to further explore differences in PRS between screen-positive and -negative patients. These results extend to the epilepsies what has previously been shown in other conditions, such as autism, that polygenic burden is elevated in cases relative to controls, even in those which carry damaging rare variants.^55^

We note that GGE PRS was not significantly increased relative to controls in any of our ‘epilepsy with ID’ cohorts, unlike the ‘DEE only’ cohorts where such an enrichment was consistently observed. A potential explanation for this difference is that a proportion of the cases which are non-DEE in the ‘epilepsy with ID’ cohort have a differing polygenic aetiology to the DEE samples in the same cohorts. This would also help explain the contrasting heterogeneity values between the the epilepsy with ID and DEE only analysis (Supplemental Figures S3 and S4). However, given we lack the phenotypic resolution to identify the DEE cases in the ‘epilepsy with ID’ cohorts, the exact cause of the differing PRS patterns remains unclear. Sample size should also be considered as a limitation in ruling out FE or GGE PRS risk factors in the corresponding DEE and ‘epilepsy with ID’ cohorts

Although the DEEs are primarily monogenic disorders, based on the results presented here we know that, while PRS only explain a small amount of phenotypic variance in each of the cohorts analysed (*R^2^* levels varying from 0.08% to 3.3%), DEEs display a clear signal of polygenicity. This could explain why pathogenic *de novo* genetic variants in DEEs can occasionally display incomplete penetrance or show marked differences in phenotypic severity.^14^^,^^56^, ^57^, ^58^ We hypothesise that genetic variants could act as modulators of the pathogenicity of highly damaging rare genetic variants or additional environmental factors, and are likely to partially explain phenotypic variation in individuals sharing a specific pathogenic variant, as has been shown for a range of other conditions.^59^ Followup work is required to determine to what extent the observed PRS signal represents a small effect in most cases of DEEs and ‘epilepsy with ID’, or rather a larger effect in particular cases, perhaps dictated by specific genes of variant type.

Our results raise further important questions, such as the role of PRS in DEE cases with pathogenic variants in the same gene. For example, do individuals with loss-of-function variants in *SCN1A* have an increased PRS for epilepsy relative to controls? Or, do PRS vary depending on the gene affected by a pathogenic variant (i.e., comparisons between carriers of variants in *SCN1A, STXBP1*, and other such genes). Do comorbid disorders or the severity of epilepsy vary according to PRS for specific traits? Larger and well-phenotyped genomic cohorts for epilepsy and other neurological disease are required to answer these questions.

At this point in time, the effect size of epilepsy-related PRS in DEEs are currently too small to be considered of value for diagnostic, treatment or prognostic purposes. This contrasts with conditions such as breast cancer, where trials are underway using PRS to identify those most at risk who are then selected for earlier mammographic screening.^60^ However, with analysis of larger datasets, PRS (and/or pTDT analysis) may add value to genetic diagnostics, when combined with rare variant analysis, potentially allowing for clinically relevant effect sizes to emerge in the context of gene-specific analyses. However, analysis of larger cohorts may show larger effect sizes, perhaps in particular genes or with certain mutation types. With larger effect sizes, PRS could potentially impact the clinic, perhaps as a prognostic guide.^61^ Most genetic research into the DEEs is currently conducted using data from exome analyses,^62^ which are not suitable for PRS calculation. Results presented here motivate the supplementation of large exome research studies with data from microarrays, as done in the Epi25 consortium.^20^^,^^21^ The move from exome to whole-genome sequencing would allow for the analysis of both common and rare genetic variants, among other benefits (such as more even sequence coverage).^63^ This research would provide value for our biological understanding of the aetiology of the DEEs, in addition to potentially explaining some of the variable presentation of DEE phenotypes.

This study has a number of weaknesses. Firstly, differences in data availability across cohorts meant that rare-variant annotation differed across all samples. The current ‘gold-standard’ approach to genetic diagnostics requires variant interpretation according to standards from the ACMG, including diagnostic deliberation with input from clinical genetics and the wider clinical team.^43^ Where possible, we applied these criteria (e.g., for the Irish Epilepsy Lighthouse and Genomics England datasets), but for most cohorts, we were limited to an *in silico* analysis. This discrepancy in pathogenicity assignment may explain the differences in the proportion of cases which screen-positive between cohorts. Additionally, although we had data from a range of projects which would be broadly representative of DEEs, or cases of ‘epilepsy with ID’, our study focused solely on individuals of European ancestry. Further work is required to verify these results in other ethnic backgrounds.

In summary, we provide evidence that DEEs harbour a polygenic component. Future studies of DEEs should look beyond monogenicity and focus on DEEs as a group of complex disorders. Large international collaborative efforts will further elucidate the complex genetic aetiology of the severe early-onset epilepsies.

## Contributors

All authors have read and approved the final draft of this manuscript.

Conceptualisation: Ciarán Campbell, Costin Leu, Gianpiero L. Cavalleri, Dennis Lal.

Investigation: The Epi4K Collaborative, The Epi25 Collaborative, Genomics England Research Consortium.

Formal analysis and Verification of Data: Ciarán Campbell, Stefan Wolking, Colin Ellis, Helena Martins, Karen Oliver, Isabelle Boothman, Claudia Moreau, Yen-Chen Anne Feng, Katherine Benson, Shiva Ganesan.

Supervision: Gianpiero Cavalleri, Norman Delanty.

Writing - original draft: Ciarán Campbell, Gianpiero L. Cavalleri.

Writing - review and editing: Norman Delanty, Patrick Cosette, Simon Girard, Sanjay Sisodiya, Ingrid E. Scheffer, Holger Lerche, Berge A. Minassian, Fadi F. Hamdan, Jacques L. Michaud, Lawrence Brody, Anne Molloy.

## Declaration of interests

Dennis Lal received research support from Taysha Therapeutics via his institution, received consulting fees from Encoded Therapeutics and honoraria for lectures from Stoke Therapeutics.

Gianpiero Cavalleri received grant funding from Janssen Pharmaceuticals and Congenica for projects not directly related to this manuscript.

Ingrid Scheffer received consulting fees from Atheneum Partners, Care Beyond Diagnosis, Epilepsy Consortium, Ovid Therapeutics, UCB, Zynerba Pharmaceuticals, honoraria for lectures or presentations from Athena Diagnostics, Biocodex, BioMarin, Chiesi, Eisai, GlaxoSmithKline, Liva Nova, UCB, support to attend meeting from Biocodex, BioMarin, Eisai, GlaxoSmithKline and UCB, has two patents WO/2006/13358 and WO/2013/059884 and a pending patent WO/2009/086591, participates on the journal board of The Lancet Neurology, Progress in Epileptic Disorders series, Epilepsy Currents, Epileptic Disorders and Neurology, is on the Scientific Advisory Board of BioMarin, Chiesi, Eisai, Encoded Therapeutics, GlaxoSmithKline, Knopp Biosciences, Takeda Pharmaceuticals, UCB, Xenon Pharmaceuticals, is the non executive director of BellberryLtd and is a trial investigator for Anavex Life Sciences, Cerebral Therapeutics, Cerecin Inc, Eisai, Encoded Therapeutics, EpiMinder Inc, Epygenyx, ES-Therapeutics, GW Pharmaceuticals, Marinus Pharmaceuticals, Neurocrine BioSciences, Ovid Therapeutics, Takeda Pharmaceuticals, UCB, Ultragenyx, Xenon Pharmaceuticals, Zogenix, Zynerba Pharmaceuticals.

Norman Delanty received grants or contracts from GW Pharma, Uneeg, IIP Novartis.

Stefan Wolking received grants or contracts from the German Research Foundation for projects not directly related to this manuscript, honoraria for speaker fees from Angelini, support for travel from Eisai and Angelini, is the president of the German Epilepsy Society (ILAE Branch) – Commission for Epilepsy and Genetics.

Yen-Chen Anne Feng is supported by the “National Taiwan University Higher Education Sprout Project (NTU-110L8810)” within the framework of the Higher Education Sprout Project by the Ministry of Education (MOE) in Taiwan.

Holger Lerche received grants from the German Research Foundation, the Federal Ministry for Education and research, Else-Kröner Fresenius Foundation, Bial, Boehringer Ingelheim for projects not directly related to this manuscript, consulting fees from Bial, Eisai, UCB/Zogenix, Arvelle/Angelini Pharma, honoraria for lectures from Bial, Eisai, UCB/Zogenix, Desitin, support for attending meetings and/or travel from Bial, Eisai, UCB/Zogenix, Desitin, participated on the data safety and monitoring board of IntraBio, chaired the Genetics Commission of the ILAE.

The other authors have no conflicts of interest to report.

## Data sharing statement

Genetic datasets underlying this project can be accessed upon the approval of a project proposal from each studies’ data access commitee.

## Appendix Supplementary materials

Image, application 1

Image, application 2
