# Supplementary material for: The role of common genetic variation in presumed monogenic epilepsies
Source: eBioMedicine. 2022 Jun 6;81:104098. doi: 10.1016/j.ebiom.2022.104098 (PMC9188960; doi:10.1016/j.ebiom.2022.104098)
Supplement: Supplementary file 1 [file mmc1.docx]

Supplemental Figures:

**Supplemental Figure S1:**

Principal component analysis (PCA) plots of cases and controls were generated independently for each analytical cohort.

S1a: PCA of Epi25 cohort. Cases are blue and controls are red.


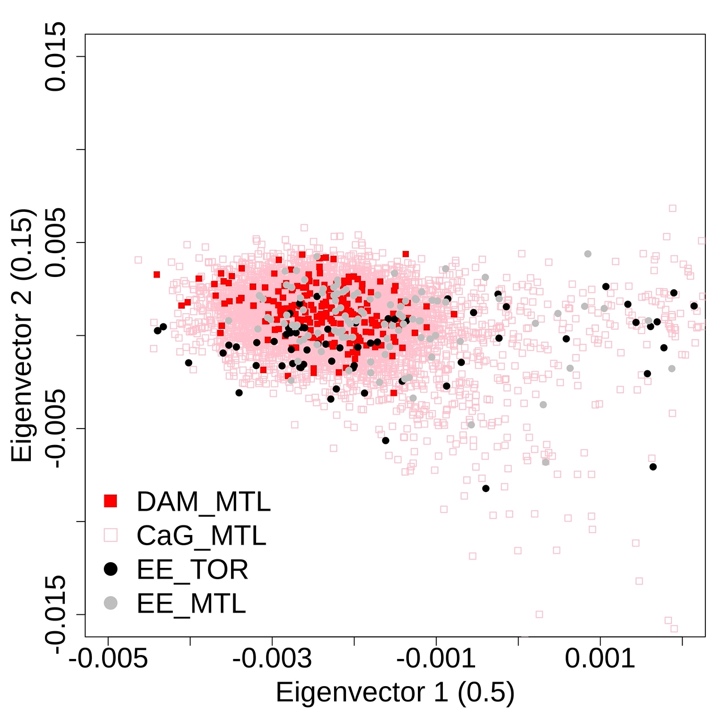


S1b: PCA of CENet cohort. Cases are black and grey circles, controls are red and pink squares.


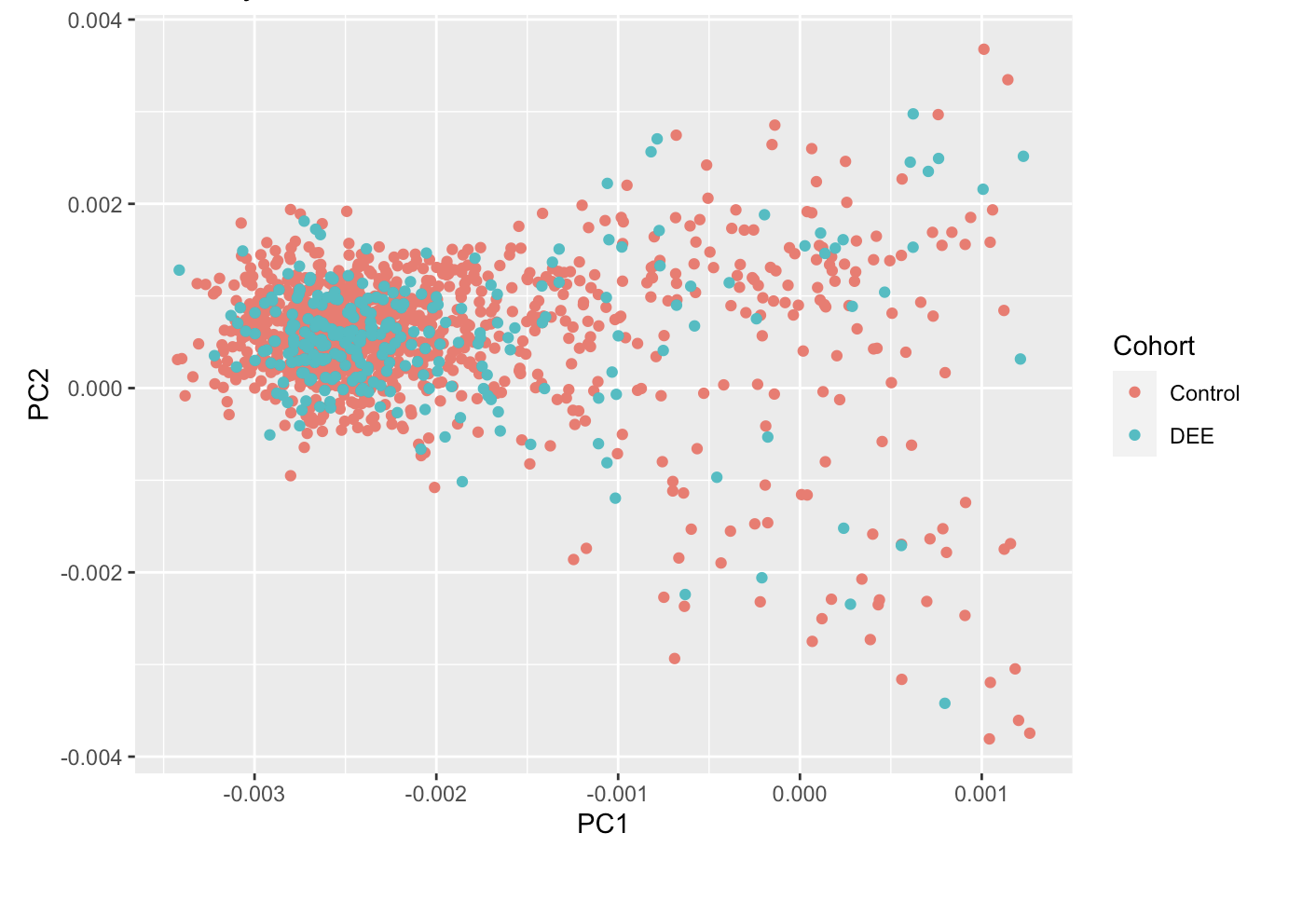


S1c: PCA of Epi4K data. Cases are blue, controls are red.

S1d: PCA of GEL cohort. Epilepsy cases are red, renal control data are blue.


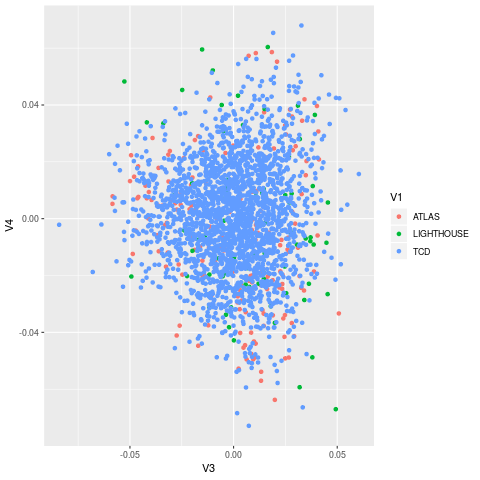


S1e: PCA of Irish Lighthouse cases shown in green with controls in blue and red.


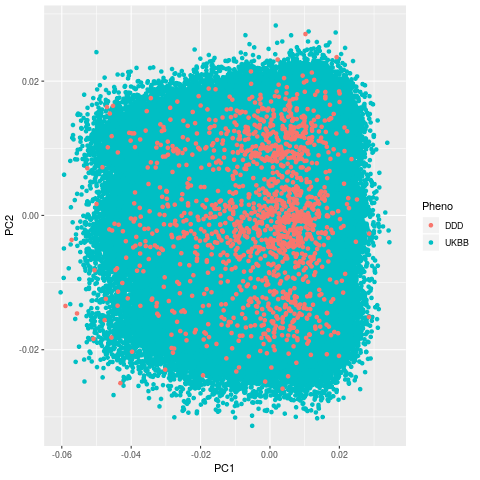


S1f: PCA of DDD cases shown in red with UK Biobank controls in blue.

| **Cohort** | **Control PRS** | **Log OR** | **SE** | **P** |
| --- | --- | --- | --- | --- |
| **EPI25** | Type 2 diabetes  (Xue et al., 2018) | -2.21E-02 | 3.15E-02 | 0.48317 |
| **CENet** | Rheumatoid arthritis (Okada et al., 2014) | 0.17 | 0.09194 | 0.06445 |
| **Epi4K** | Asthma  (Demenais et al., 2018) | 0.09864 | 0.0636 | 0.1209 |
| **Lighthouse** | Rheumatoid arthritis  (Okada et al., 2014) | -0.1735 | 0.1126 | 0.1233 |
| **DDD** | Asthma  (Demenais et al., 2018) | -0.03346 | 0.02673 | 0.2106 |
| **GEL** | Rheumatoid arthritis  (Stahl et al., 2010) | 0.08596 | 0.06758 | 0.203 |

**Table S1:** Logistic regression comparing control PRS of unrelated traits between cases and controls in each cohort. No significant associations were observed

| **Test** | **All Epi PRS** | | | **Focal PRS** | | | **GGE PRS** | | |
| --- | --- | --- | --- | --- | --- | --- | --- | --- | --- |
|  | Log OR | SE | P | Log OR | SE | P | Log OR | SE | P |
| **Controls – screen-negative** | 0.1098 | 0.0211 | <0.0001 | 0.1327 | 0.0207 | <0.0001 | 0.0744 | 0.0214 | 0.0005 |
| **Controls – screen-positive** | 0.1480 | 0.0448 | 0.001 | 0.1844 | 0.0441 | <0.0001 | 0.0006 | 0.0445 | 0.9886 |
| **Screen-negative – screen-positive** | 0.0100 | 0.0495 | 0.8394 | 0.0223 | 0.0276 | 0.4186 | -0.0747 | 0.0511 | 0.1436 |

**Table S2:** Fixed-effects meta-analyses comparing cases with or without likely deleterious genetic variants to each other and population controls. Log OR = Log odds ratio, SE = standard error, P = p-value.

| **Test** | **All epilepsy** | **Focal** | **Generalised** |
| --- | --- | --- | --- |
| **Case / Control** | 83.31% | 85.57% | 84.03% |
| **Screen Positive / Control** | 61.10% | 77.24% | 45.12% |
| **Screen Negative / Control** | 46.49% | 83.19% | 50.32% |
| **Screen Positive / Screen Negative** | 0.00% | 0.00% | 0.00% |

**Table S3:** Showing *I^2^* values for all meta-analyses conducted.

**
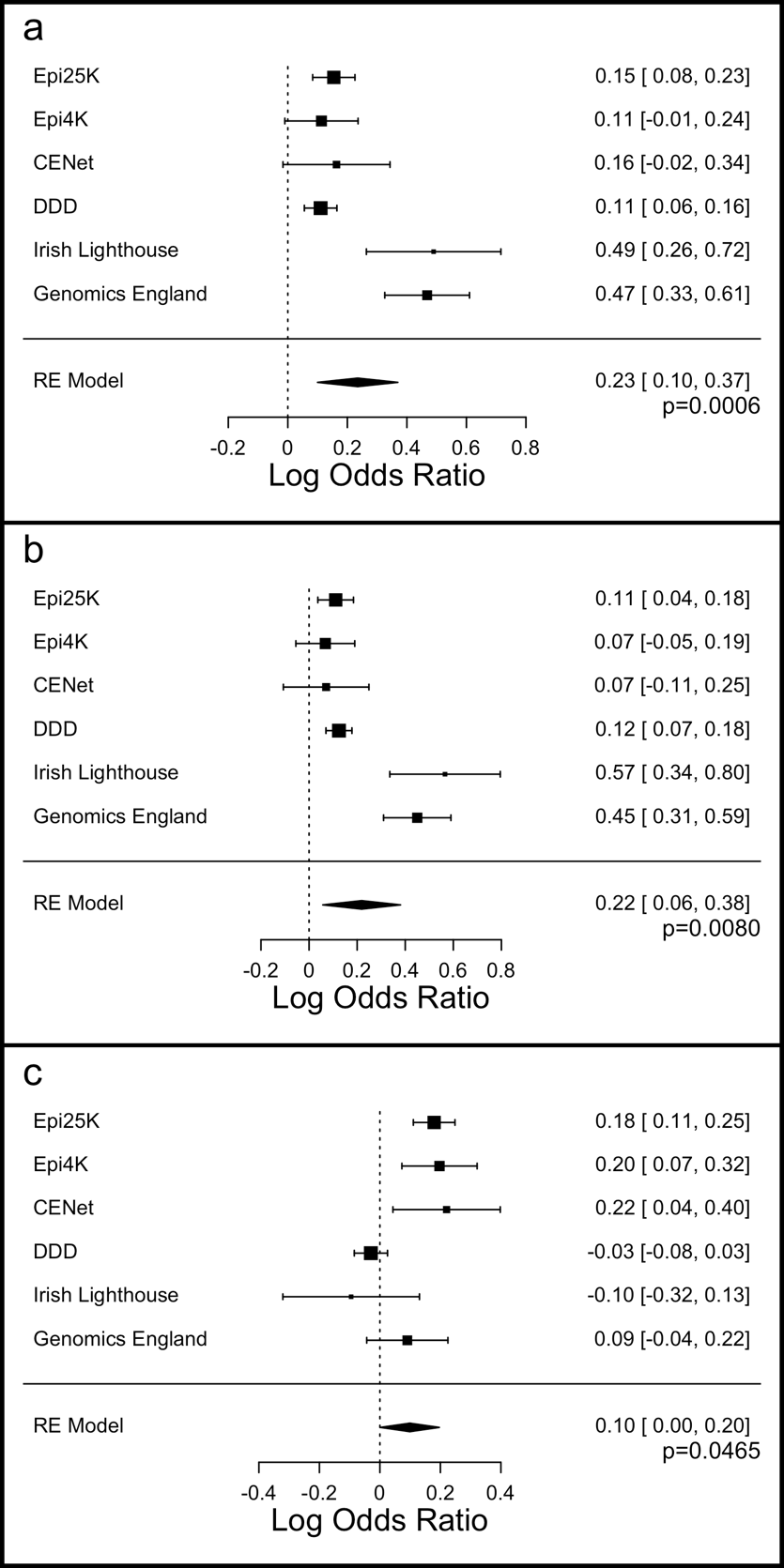
**

**Figure S2**: Meta-analysis of PRS of a) ‘all epilepsy’, b) ‘Focal epilepsy’, and c) GGE conducted using a random effects model. ‘RE Model’ = Random-effects model. Box plots show log odds ratios and standard errors. ‘epi + ID’ refers to epilepsy with intellectual disability.


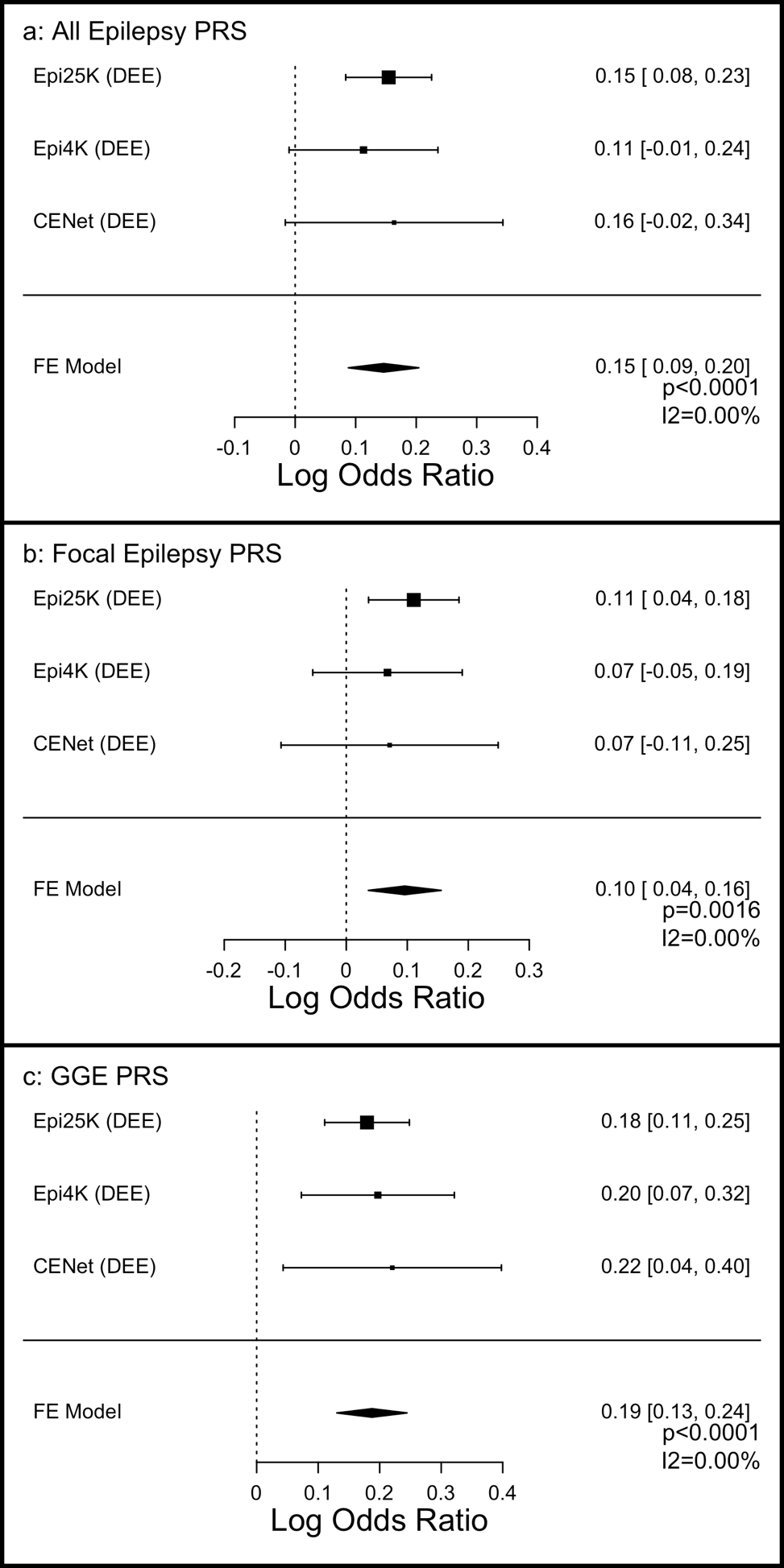


**Figure S3:** Meta-analysis of PRS of a) ‘all epilepsy’, b) ‘Focal epilepsy’, and c) GGE conducted using a fixed effects model on developmental and epileptic encephalopathies (DEE) cohorts only. ‘FE Model’ = Fixed-effects model. Box plots show log odds ratios and standard errors. Also shown are p-values and *I^2^* values for each meta-analysis.

**
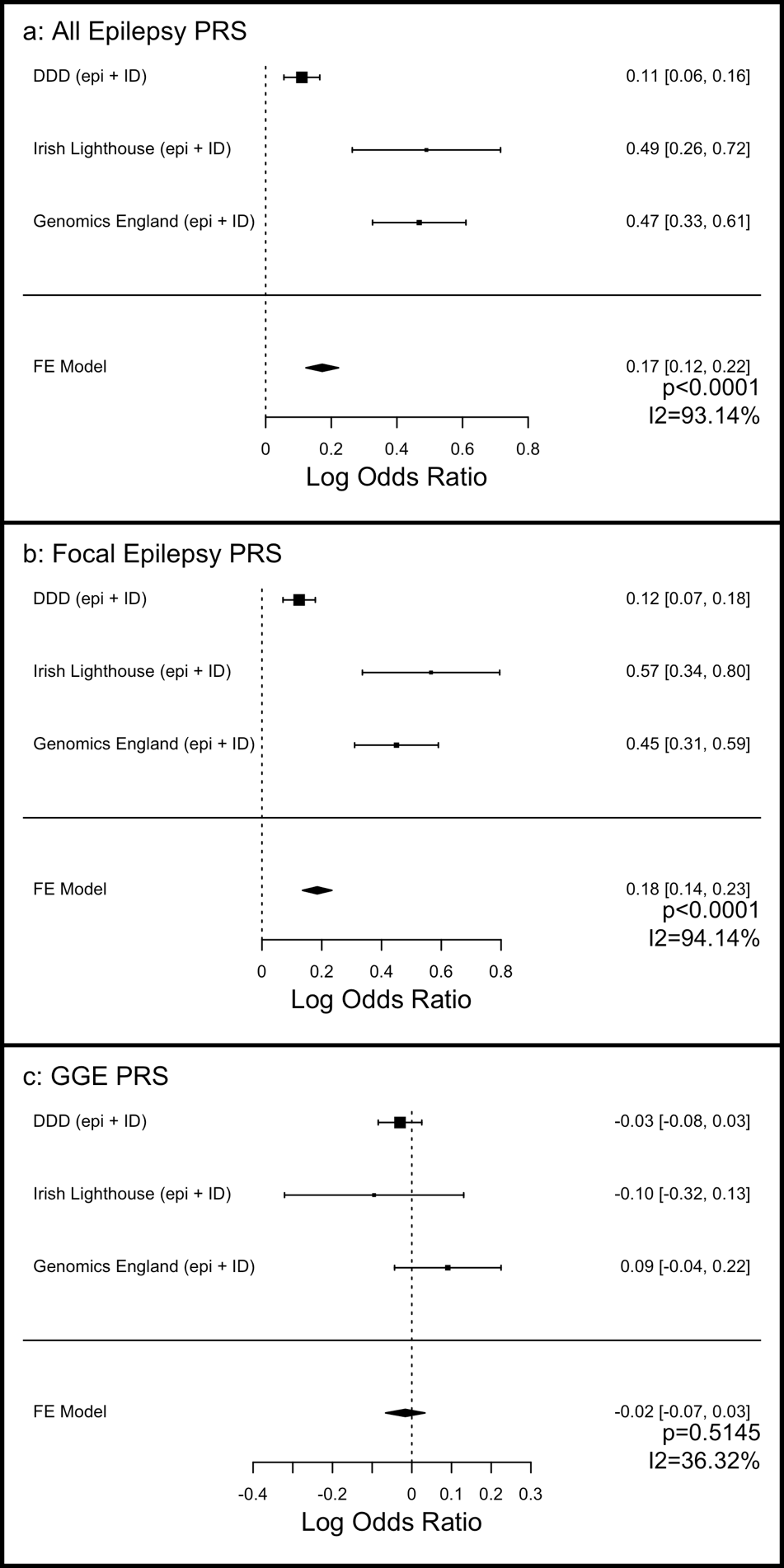
**

**Figure S4:** Meta-analysis of PRS of a) ‘all epilepsy’, b) ‘Focal epilepsy’, and c) GGE conducted using a fixed effects model on epilepsy with ID (‘epi +ID’) samples only. ‘FE Model’ = Fixed-effects model. Box plots show log odds ratios and standard errors. Also shown are p-values and *I^2^* values for each meta-analysis.
