# Supplementary material for: The role of common genetic variation in presumed monogenic epilepsies
Source: eBioMedicine. 2022 Jun 6;81:104098. doi: 10.1016/j.ebiom.2022.104098 (PMC9188960; doi:10.1016/j.ebiom.2022.104098)
Supplement: Supplementary file 2 [file mmc2.docx]

**Supplemental Figure S1a:**

**Title**: PCA of Epi25 cohort.

**Legend**: Principal component analysis (PCA) plots of cases and controls were generated independently for each analytical cohort. Cases are blue and controls are red.

**Supplemental Figure S1b:**

**Title**: PCA of CENet cohort.

**Legend**: Cases are black and grey circles, controls are red and pink squares.

**Supplemental Figure S1c:**

**Title**: PCA of Epi4K data.

**Legend**: Cases are blue, controls are red.

**Supplemental Figure S1d:**

**Title**: PCA of GEL cohort.

**Legend**: Epilepsy cases are red, GEL renal control data are blue.

**Supplemental Figure S1e:**

**Title**: PCA of Irish Lighthouse cases and controls

**Legend**: cases are shown in green, with controls in blue and red.

**Supplemental Figure S1f:**

**Title**: PCA of DDD cases and UK Biobank controls

**Legend**: cases are shown in red with UK Biobank controls in blue.

**Table S1:**

**Title**: Logistic regression comparing control PRS of unrelated traits between cases and controls in each cohort. No significant associations were observed

**Table S2**

**Title:** Fixed-effects meta-analyses comparing cases with or without likely deleterious genetic variants to each other and population controls.

**Legend:** Log OR = Log odds ratio, SE = standard error, P = p-value.

**Table S3:**

**Title:** Showing *I^2^* values for all meta-analyses conducted.

**Figure S2**: Meta-analysis of PRS of a) ‘all epilepsy’, b) ‘Focal epilepsy’, and c) GGE conducted using a random effects model. ‘RE Model’ = Random-effects model. Box plots show log odds ratios and standard errors. ‘epi + ID’ refers to epilepsy with intellectual disability.

**Figure S3:** Meta-analysis of PRS of a) ‘all epilepsy’, b) ‘Focal epilepsy’, and c) GGE conducted using a fixed effects model on developmental and epileptic encephalopathies (DEE) cohorts only. ‘FE Model’ = Fixed-effects model. Box plots show log odds ratios and standard errors. Also shown are p-values and *I^2^* values for each meta-analysis.

**Figure S4:** Meta-analysis of PRS of a) ‘all epilepsy’, b) ‘Focal epilepsy’, and c) GGE conducted using a fixed effects model on epilepsy with ID (‘epi +ID’) samples only. ‘FE Model’ = Fixed-effects model. Box plots show log odds ratios and standard errors. Also shown are p-values and *I^2^* values for each meta-analysis.
